# Supplementary material for: Treatment effects in epilepsy: a mathematical framework for understanding response over time
Source: Front Netw Physiol. 2024 Jun 26;4:1308501. doi: 10.3389/fnetp.2024.1308501 (PMC11233745; doi:10.3389/fnetp.2024.1308501)
Supplement: Supplementary file 1 [file DataSheet1.PDF]

Supplementary Material

1 SUPPLEMENTARY TABLES AND FIGURES

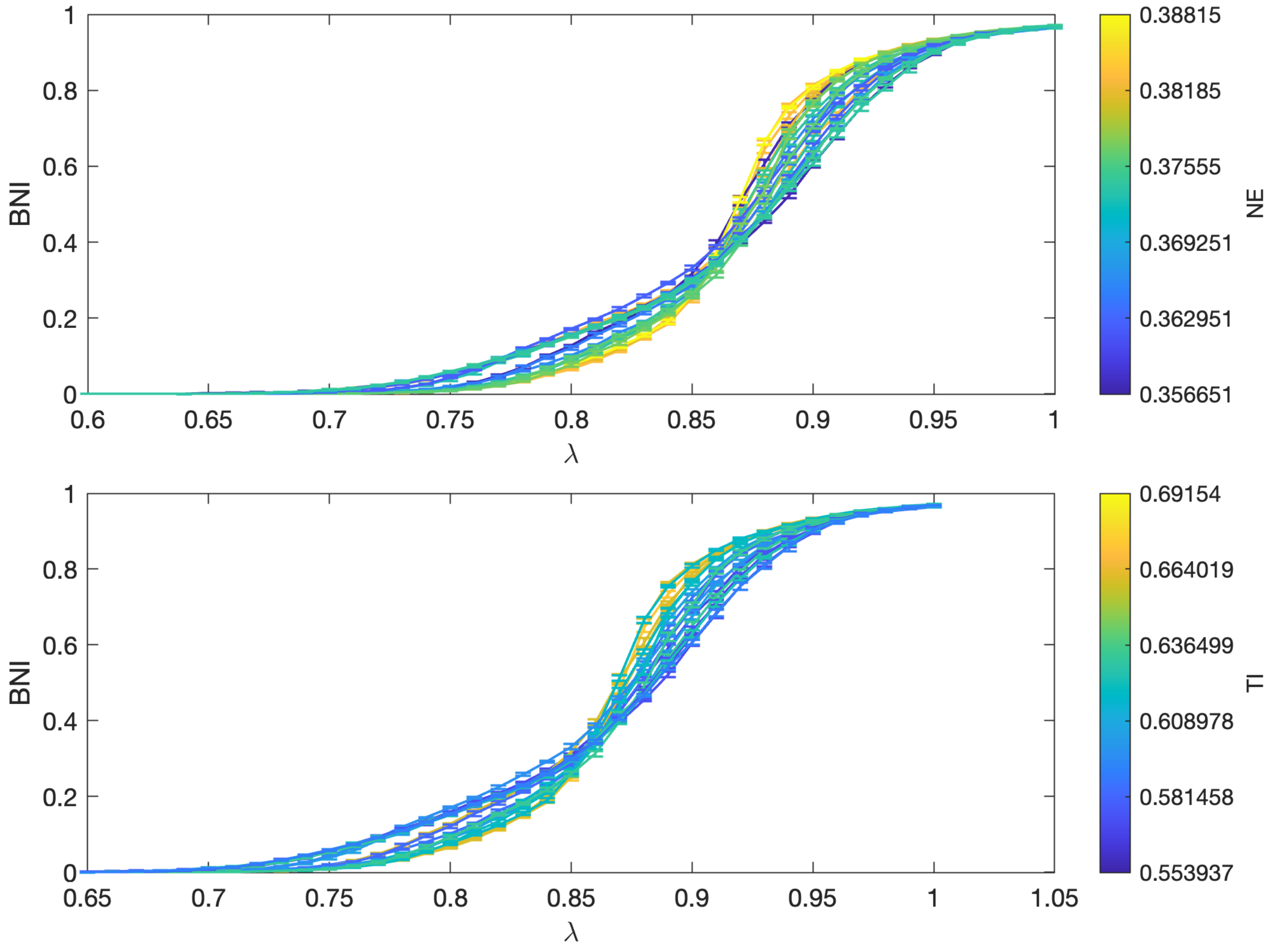

Figure S1. Increase of BNI as a function of λ_0 for 20 node networks with size of FTC, $n = 1$ and a mean degree of 2.5. Network trajectories are coloured according to their efficiency (top) and trophic incoherence (bottom).

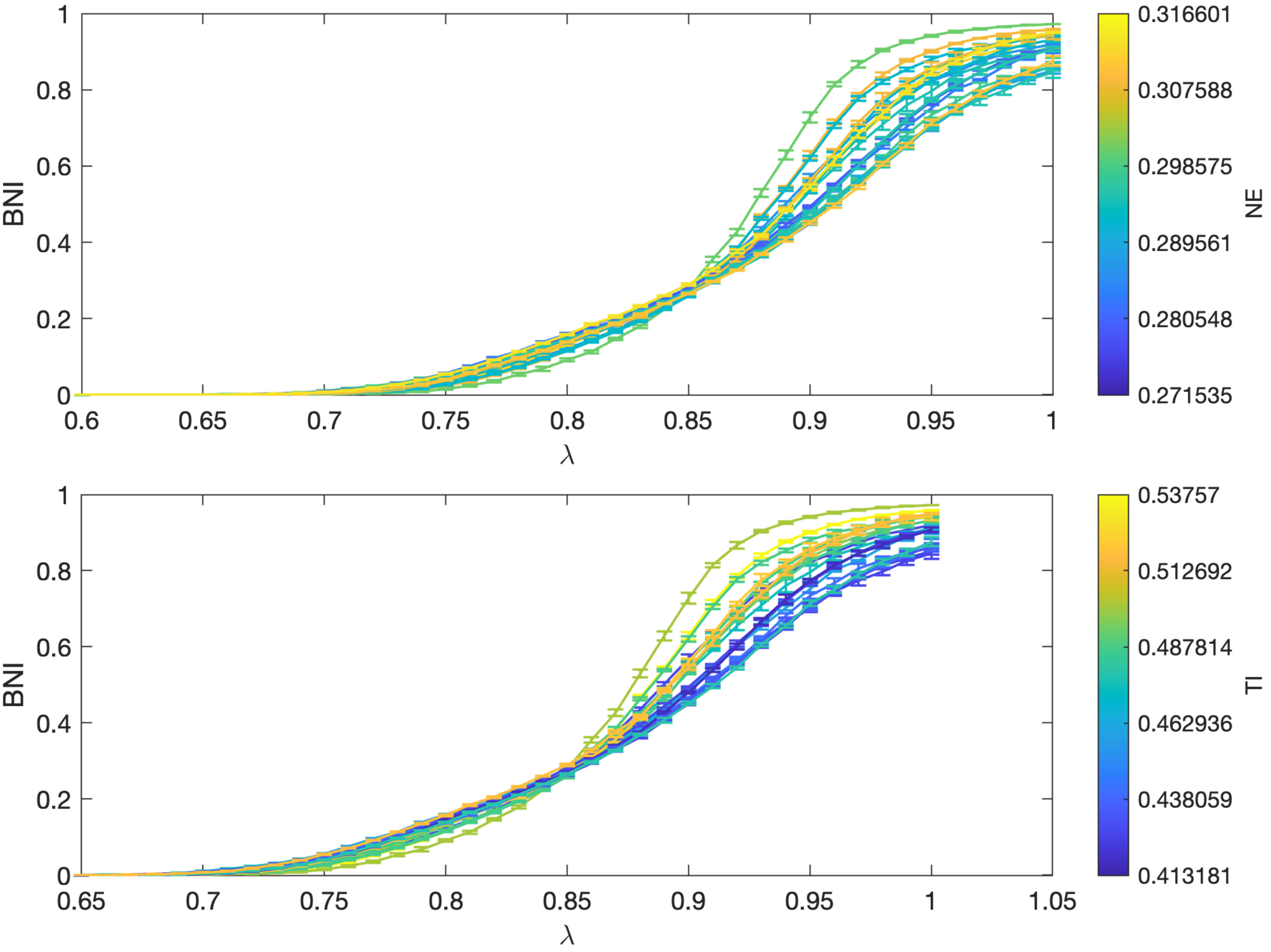

Figure S2. Increase of BNI as a function of λ_0 for 20 node networks with size of FTC, $n = 5$ and a mean degree of 2.5. Network trajectories are coloured according to their efficiency (top) and trophic incoherence (bottom).

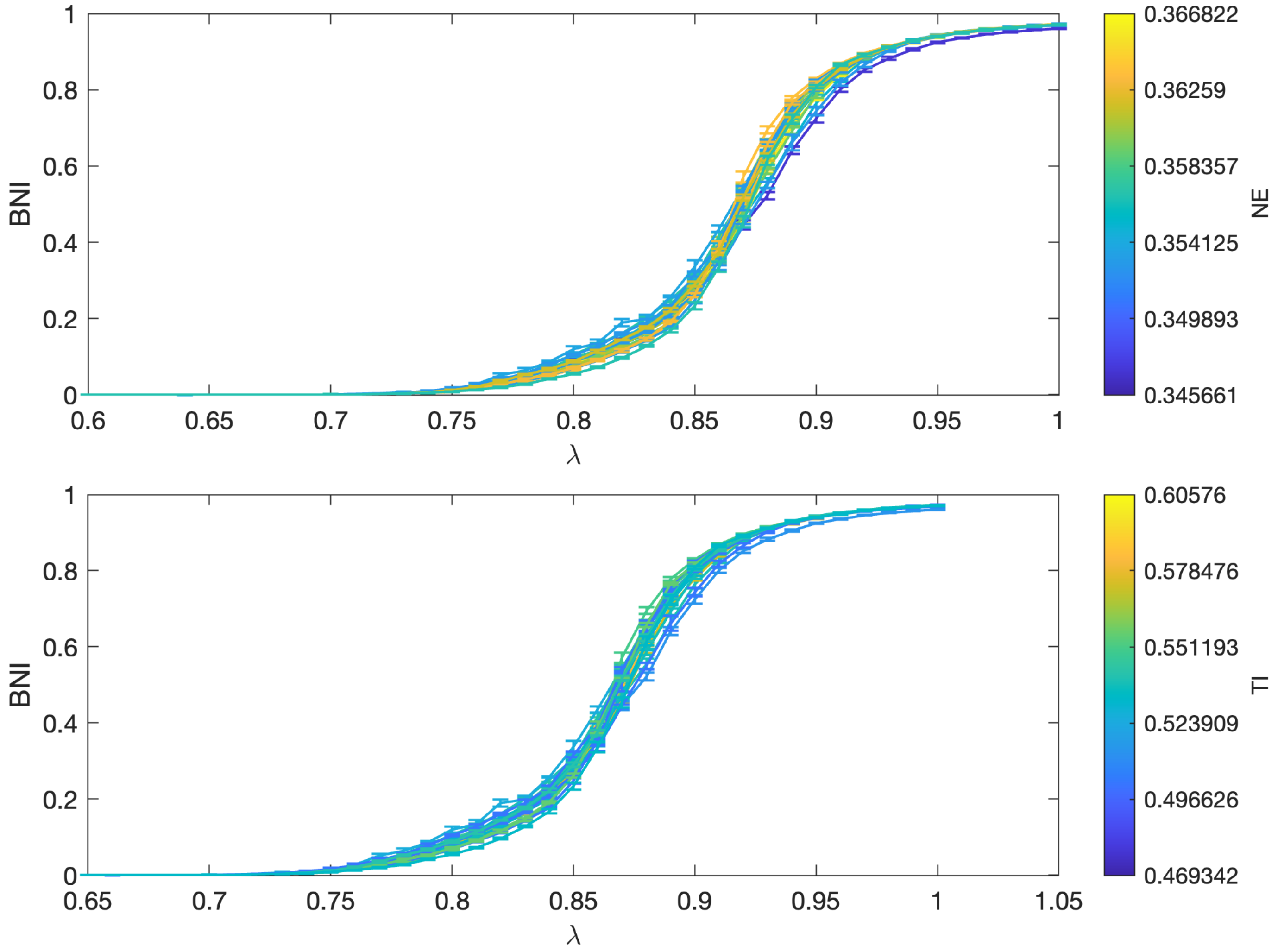

Figure S3. Increase of BNI as a function of λ_0 for 20 node networks with size of FTC, n = 16 and a mean degree of 2.5. Network trajectories are coloured according to their efficiency (top) and trophic incoherence (bottom).

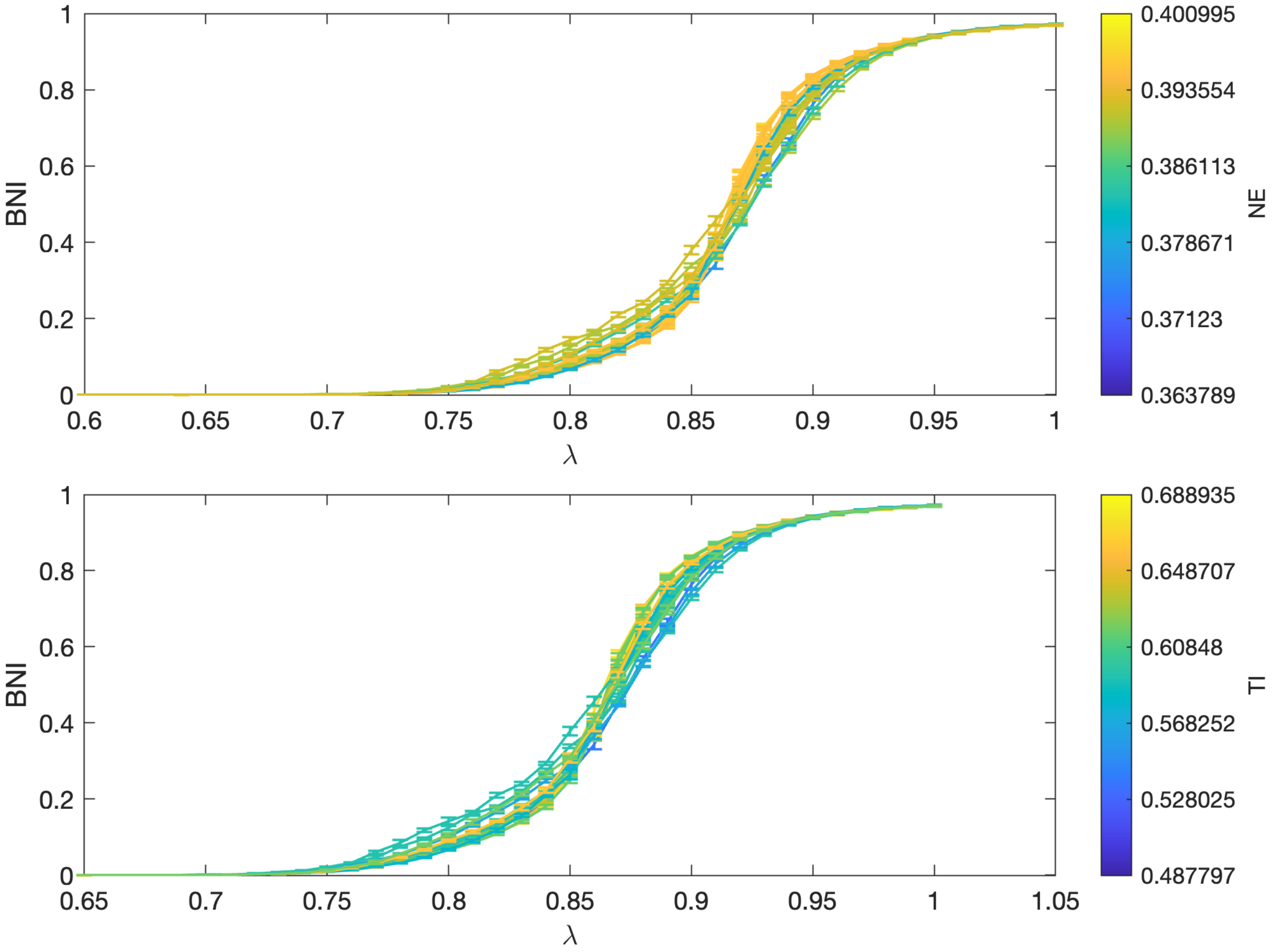

Figure S4. Increase of BNI as a function of λ_0 for 20 node networks with size of FTC, n = 20 and a mean degree of 2.5. Network trajectories are coloured according to their efficiency (top) and trophic incoherence (bottom).

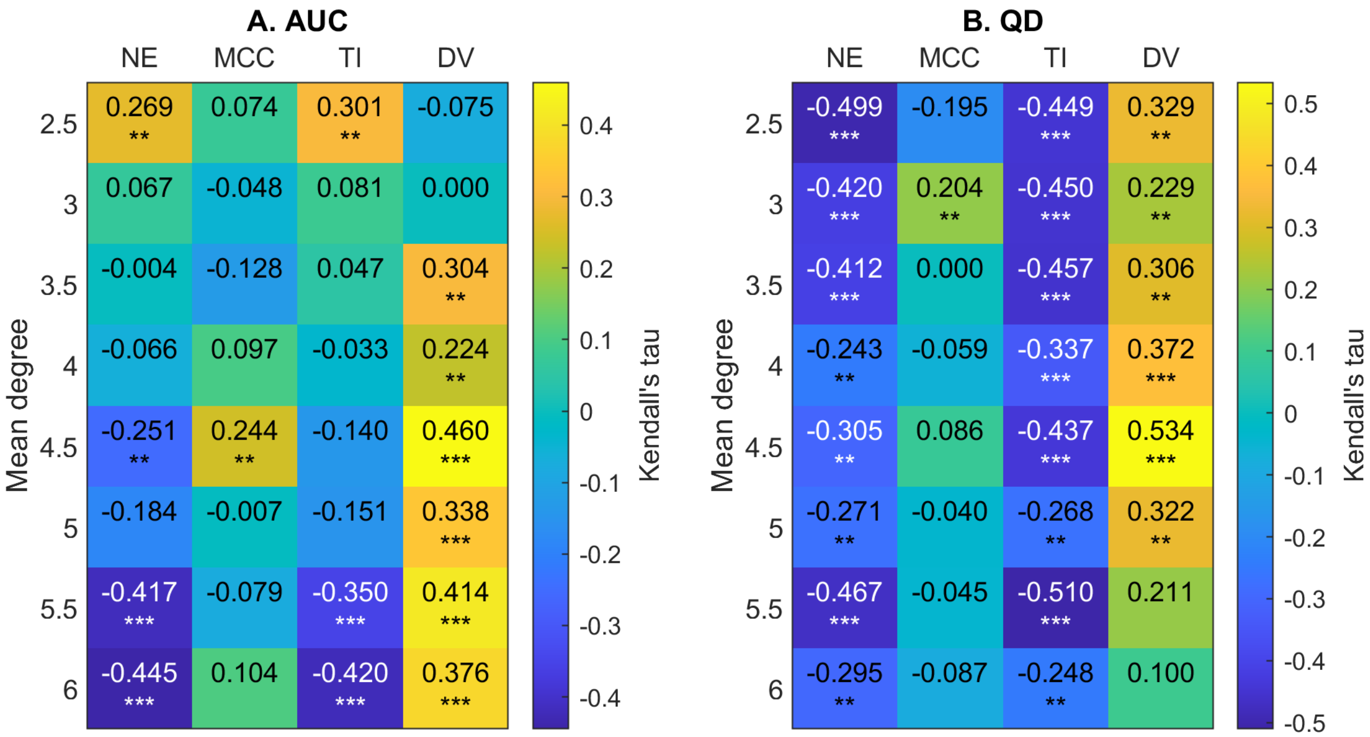

Figure S5. Heat map of Kendall correlation coefficients between network metrics (Efficiency, mean clustering coefficient, trophic incoherence and degree variance) and AUC, QD for 20 node networks generated with a increasing mean degree. A set of 50 networks with size of FTC, $n = 50$ were analysed for each value of mean degree. Significance values are labelled as * where $p < 0.05$, ** where $p < 0.01$ and *** where $p < 0.0001$.

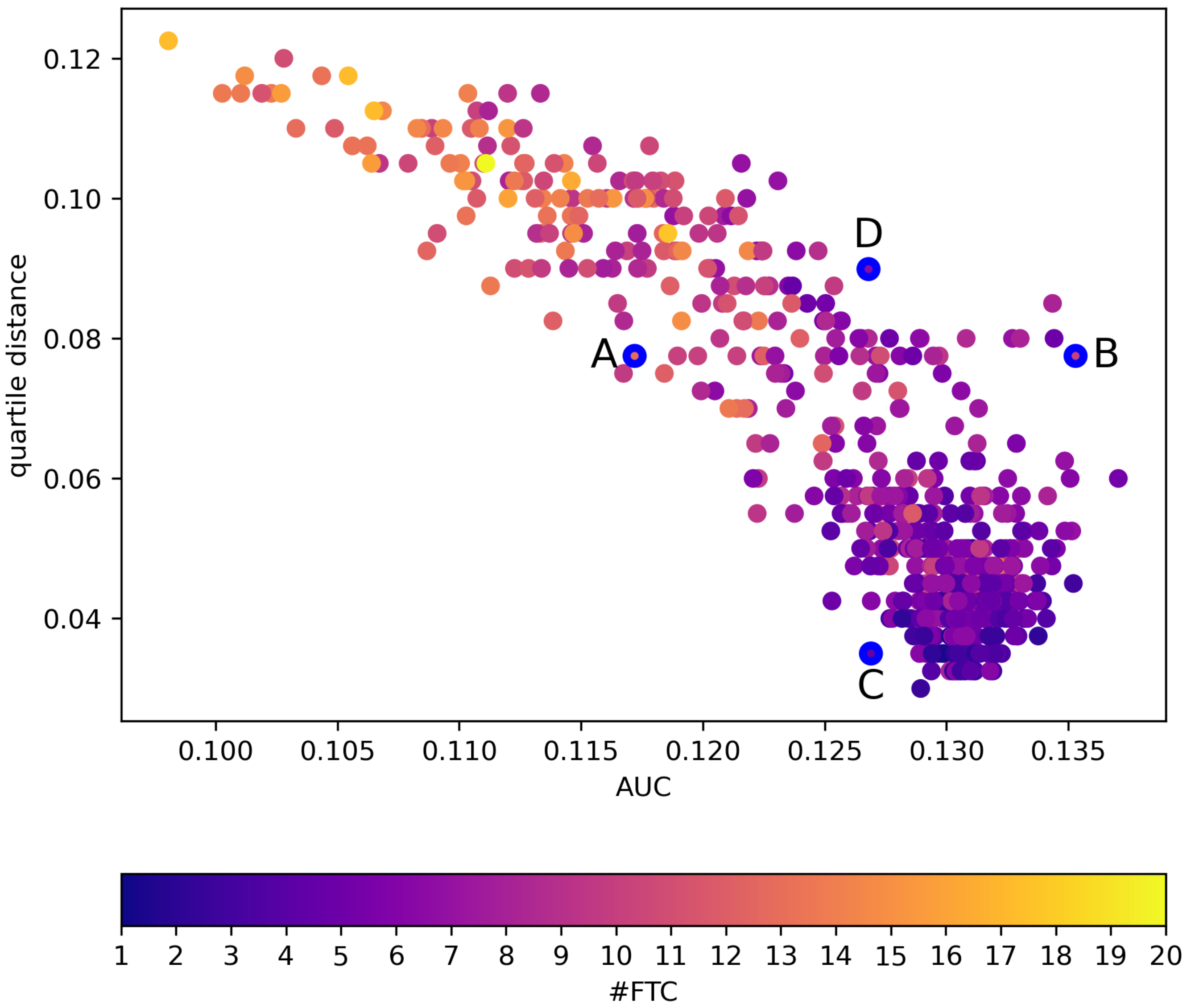

Figure S6. Scatter plot representing the relationship between QD and AUC, also displaying size of FTC on a colour scale. There is a significant negative correlation between the two (Kendall tau = -0.581, $p < 0.0001$). Points labelled A and B have a similar QD but a significantly different AUC, and points labelled C and D have a similar AUC but significantly different QDs. The differences between the λ_0-BNI trajectories of these networks are displayed in Figure S9.

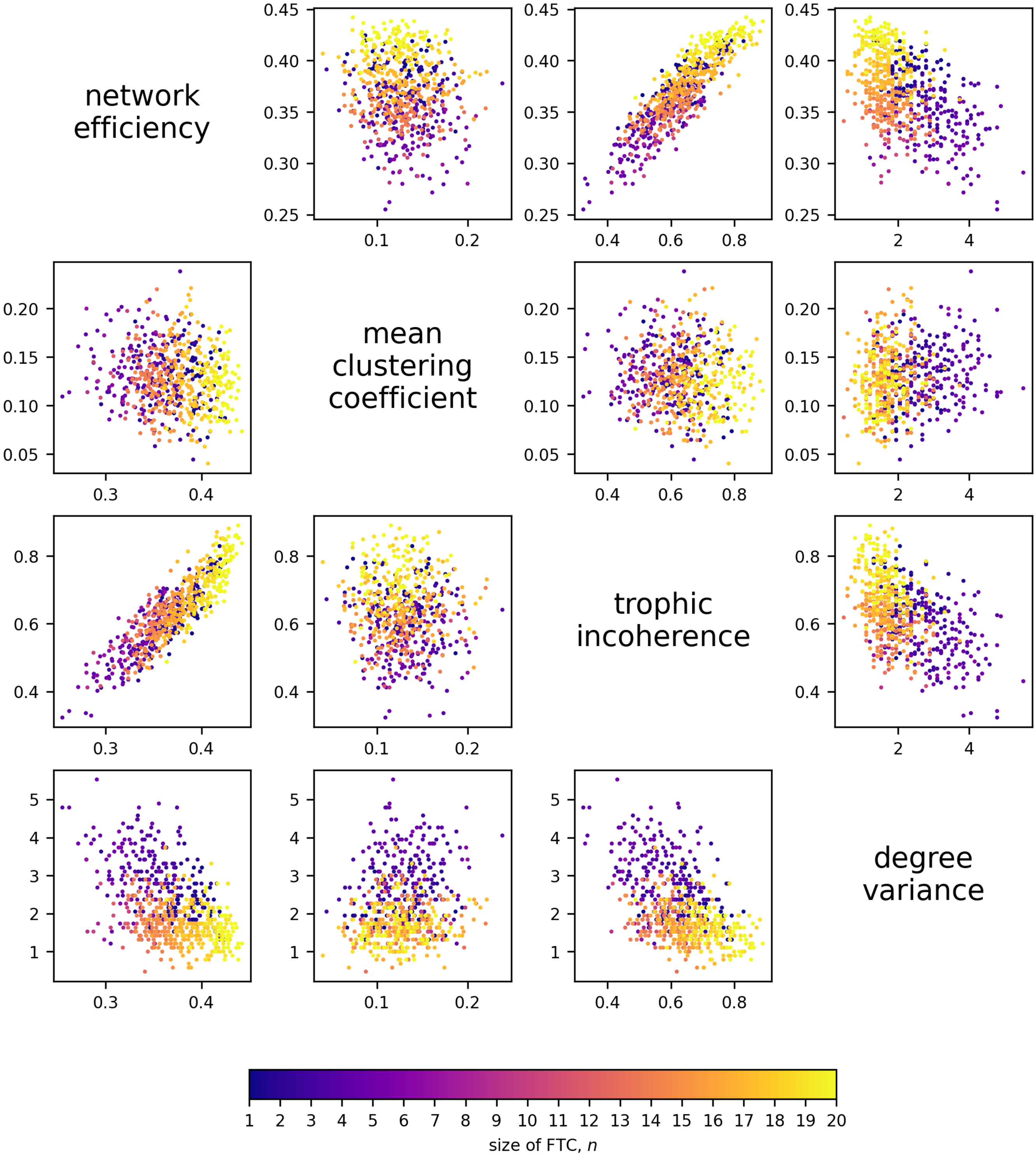

Figure S7. Plot detailing the relationships between network efficiency, mean clustering coefficient, trophic incoherence and degree variance in 20-node networks.

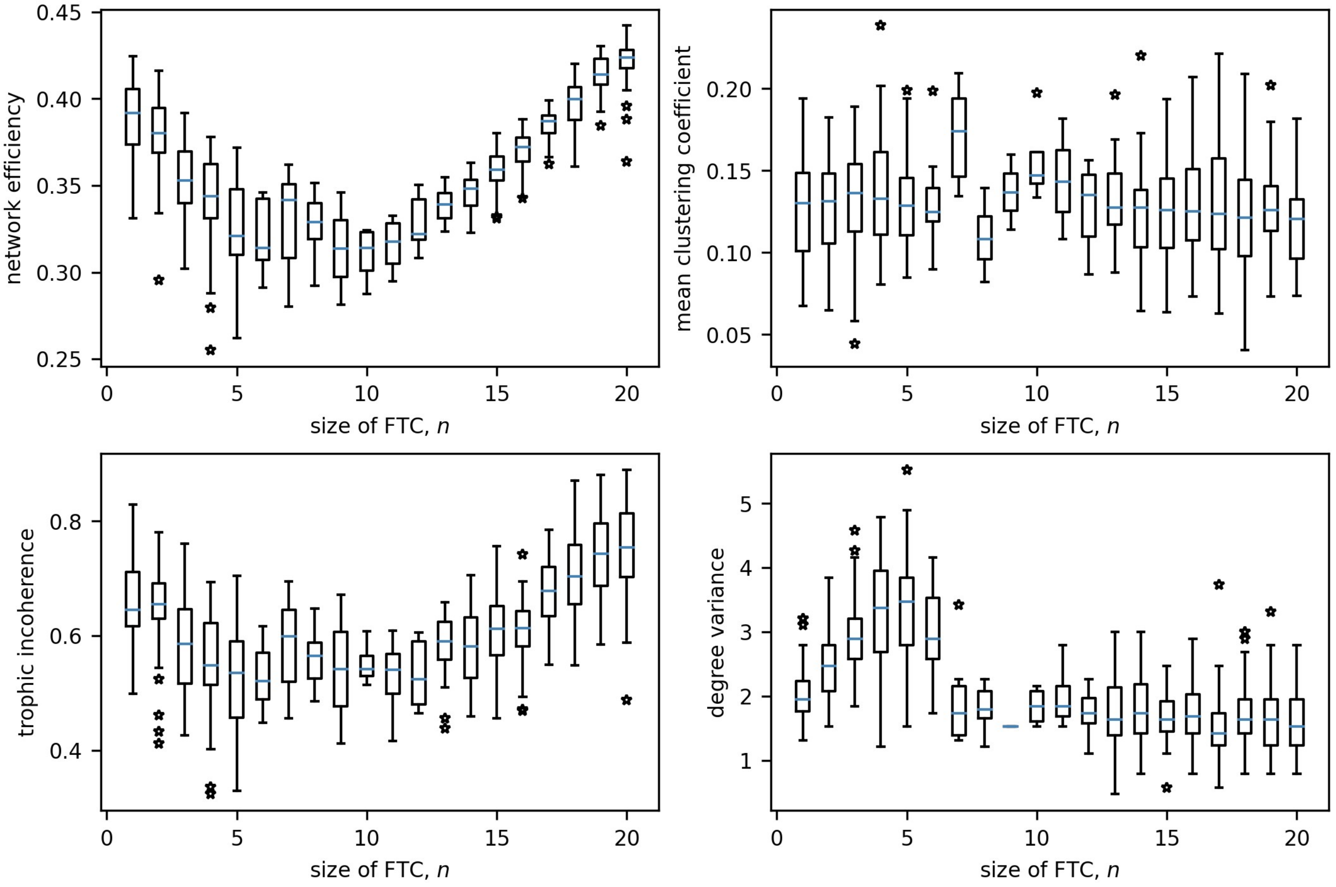

Figure S8. Boxplots detailing the variation of different network measures for each size of FTC in 20-node networks.

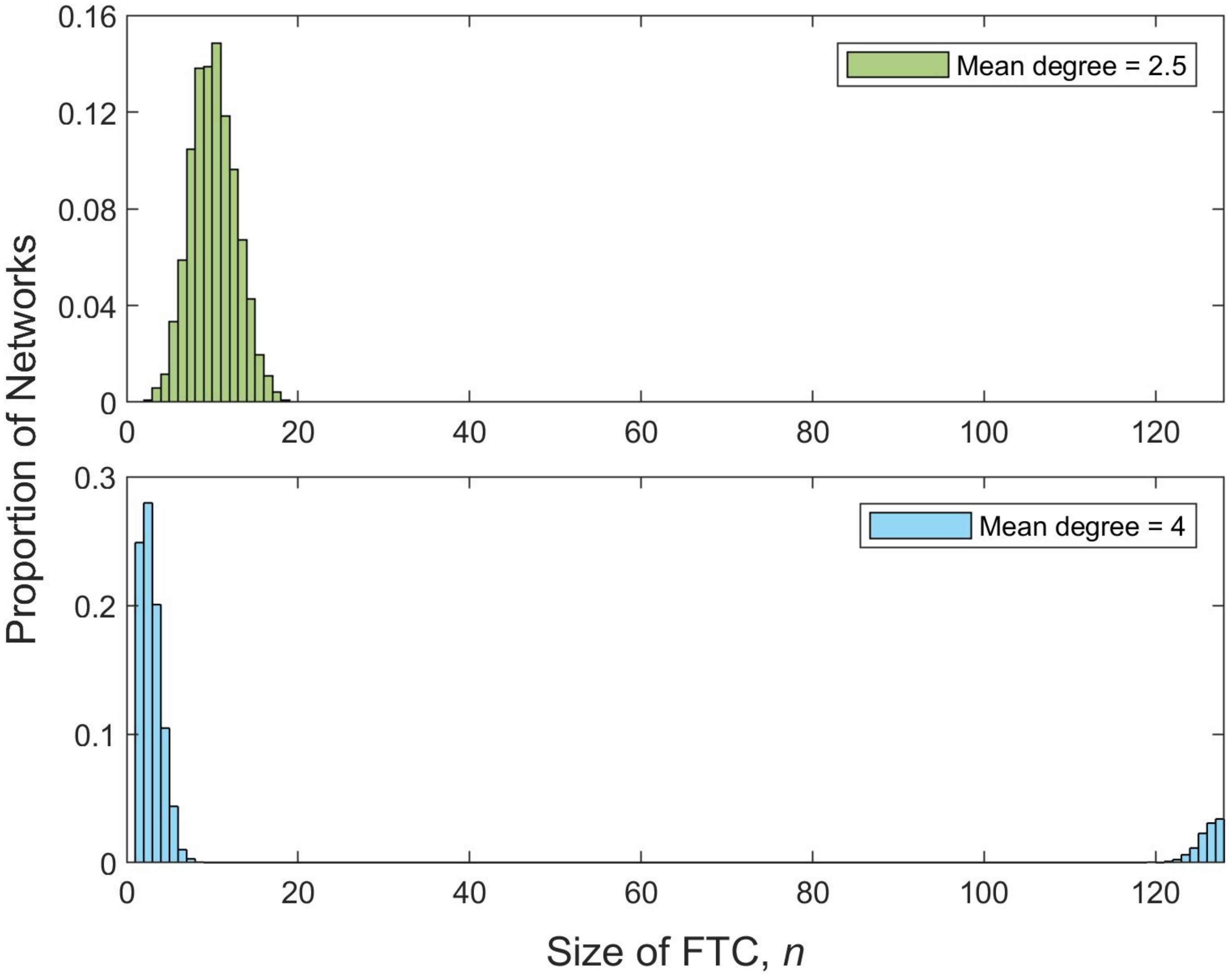

Figure S9. Sizes of FTC's of 5,000 randomly-generated 128-node networks of mean degree 2.5 and 4.
